# Pathogenesis of hemorrhagic disease caused by elephant endotheliotropic herpesvirus (EEHV) in Asian elephants (*Elephas maximus*)

**DOI:** 10.1038/s41598-021-92393-8

**Published:** 2021-06-21

**Authors:** Thunyamas Guntawang, Tidaratt Sittisak, Varankpicha Kochagul, Saralee Srivorakul, Kornravee Photichai, Kittikorn Boonsri, Thittaya Janyamethakul, Khajohnpat Boonprasert, Warangkhana Langkaphin, Chatchote Thitaram, Kidsadagon Pringproa

**Affiliations:** 1 Department of Veterinary Biosciences and Veterinary Public Health, Faculty of Veterinary Medicine, Chiang Mai University, Chiang Mai, 50100 Thailand; 2 Veterinary Diagnostic Laboratory, Faculty of Veterinary Medicine, Chiang Mai University, Chiang Mai, 50100 Thailand; 3 Patara Elephant Farm, Hang Dong, Chiang Mai, 50230 Thailand; 4 Center of Excellence in Elephant and Wildlife Research, Chiang Mai University, Chiang Mai, 50100 Thailand; 5 National Elephant Institute, Forest Industry Organization, Lampang, 52190 Thailand; 6 Department of Companion Animals and Wildlife Clinics, Faculty of Veterinary Medicine, Chiang Mai University, Chiang Mai, 50100 Thailand

**Keywords:** Pathogenesis, Infection

## Abstract

Elephant endotheliotropic herpesvirus-hemorrhagic disease (EEHV-HD) is an acute fatal disease in elephants. Despite the fact that the underlying pathogenesis of EEHV-HD has been proposed, it remains undetermined as to what mechanisms drive these hemorrhagic and edematous lesions. In the present study, we have investigated and explained the pathogenesis of acute EEHV-HD using blood profiles of EEHV-HD and EEHV-infected cases, hematoxylin and eosin (H&E) stain, special stains, immunohistochemistry, quantitative polymerase chain reaction (PCR) and reverse transcriptase polymerase chain reaction (RT-PCR). It was found that EEHV genomes were predominantly detected in various internal organs of EEHV-HD cases. Damage to endothelial cells, vasculitis and vascular thrombosis of the small blood vessels were also predominantly observed. Increases in platelet endothelial cell adhesion molecules-1 (PECAM-1)- and von Willebrand factor (vWF)-immunolabeling positive cells were significantly noticed in injured blood vessels. The expression of pro-inflammatory cytokine mRNA was significantly up-regulated in EEHV-HD cases when compared to EEHV-negative controls. We have hypothesized that this could be attributed to the systemic inflammation and disruption of small blood vessels, followed by the disseminated intravascular coagulopathy that enhanced hemorrhagic and edematous lesions in EEHV-HD cases. Our findings have brought attention to the potential application of effective preventive and therapeutic protocols to treat EEHV infection in Asian elephants.

## Introduction

Elephant endotheliotropic herpesvirus-hemorrhagic disease (EEHV-HD) is the most consequential viral infectious disease in young Asian elephants (*Elephas maximus*)^1–3^. Elephant calves that have died due to EEHV-HD displayed one or more of the following clinical signs: fever, lethargy, bloody diarrhea, facial edema or a cyanotic tongue^1, 3, 4^. Hematological evaluation of EEHV-HD calves is usually diagnosed by the emergence of anemia, thrombocytopenia, monocytopenia and/or a reduction in plasma protein concentration^1, 5^. Gross and microscopic findings can reveal generalized hemorrhages and edema of the subcutaneous, subserosa and certain internal organs including the heart, lungs, liver, spleen, kidneys, lymph nodes and intestines^2, 4^. To date, despite the fact that the mechanisms of hemorrhages and edema in EEHV-HD cases have been hypothesized^6–8^, to the best of our knowledge no such studies have investigated the pathogenesis underlying of those pathological lesions. In fact, the pathogenesis of hemorrhages, thrombocytopenia and protein leaks emitted from blood vessels during acute fatal EEHV infection are still far from being fully understood.

Mechanisms of virus-induced plasma leakage emitted from blood vessels can be due to either direct lytic endothelial cell infection or induction of physiological mechanisms that increase vascular permeability^9^. An increase in vascular permeability is often initiated in response to various cellular mediators that are secreted by the endothelia, leukocytes or perivascular mast cells^10^. On the other hand, damage to the endothelia is known to increase the expression of certain glycoproteins, such as the von Willebrand factor (vWF) and platelet endothelial cell adhesion molecules (PECAM), on the endothelia and platelets that are known to promote cell-endothelial adhesion and platelet activation^11, 12^. The acquisition of the latter may eventually cause over consumption of platelets which can then lead to disseminated intravascular coagulation (DIC). This will potentially cause irreversible vascular damage and likely result in organ dysfunction or hypovolemic shock^13, 14^.

During viral infections, platelet activation is triggered by viruses and can modulate the number of platelets by decreasing platelet production, increasing platelet consumption or increasing platelet destruction^15, 16^. All of which would depend upon the specific viral species in question. In fact, it can be stated that during the viremia phase of some virus infections, an enhancement in platelet consumption is observed as a form of virus-induced DIC^16, 17^. This would be especially true for illnesses associated with viral hemorrhagic fevers such as Dengue hemorrhagic fever, Chikungunya, Ebola, Marburg hemorrhagic fever and Lassa fever^16^. Meanwhile, an increase in platelet destruction was predominantly observed in certain virus-induced immune mediated diseases such as human immunodeficiency virus (HIV)-1^18^. On the other hand, an increase in vascular permeability by perivascular mast cells is shown to be one of the mechanisms that can induce vascular leakage in cases of Dengue hemorrhagic fever^19–21^. In elephants, it remains undetermined as to whether EEHV infection can induce vascular hyperpermeability. However, it cannot be ruled out since some elephant calves that have died as a result of acute EEHV-HD showed only a mild to moderate degree of vascular inflammation^6^. Moreover, it is of significant interest to demonstrate how EEHV infection is associated with injuries of the blood vessels and thrombocytopenia in EEHV-HD cases, which subsequently can result in bleeding disorders and may lead to severe multifocal hemorrhagic lesions in EEHV-HD calves. In the present study, we seek to investigate whether and how endothelial lesions, through the activation of platelets and the expression of cytokine, play a role in the pathomechanisms of hemorrhagic disease caused by EEHV in Asian elephants.

## Results

### Thrombocytopenia is a predictive hematological parameter for acute fatal EEHV-HD

The archival records of fatal EEHV-HD and non-fatal EEHV-infected elephant blood samples submitted to the Veterinary Diagnostic Center, Chiang Mai University between the years of 2015 and 2020 clearly indicate that thrombocytopenia was the only significant hematological parameter observed in acute fatal EEHV-HD cases when compared to either the reference values (Table 1) or the EEHV-negative controls (Table S1). This was especially true for animals aged less than 5 years old. It should be noted that despite the fact that a reduction in the platelet count was observed in fatal EEHV-HD subjects, other hematological parameters, including red blood cell counts, pack cell volume (PCV) or plasma proteins, were included in the normal reference values (Table 1).

**Table 1:** History and blood profile of fatal EEHV-HD and non-fatal EEHV-infected elephants, Chiang Mai, Thailand between the years 2015 and 2020.

Criteria	Parameters	Genotypes^b^			Ref. range^a^
		EEHV1A	EEHV4	EEHV1A/4	
Fatal EEHV-HD	No. of animals	7	2	1	
	Mean age (year-old)	2.78	3	5	
	Mean RBC count (× 10^6^ cells/µL)	2.81	3.85	4.73	2.5–5
	Mean PCV (%)	31.98	42.5	55	30–40
	Mean platelet count (× 10^3^ cells/µL)	**93.11**	**70.5**	**173**	200–600
	Mean total serum protein (g/dL)	NA	5.5	7.1	6–8
	Albumin (g/dL)	NA	2.6	2.9	1.5–3.5
	Globulin (g/dL)	NA	2.9	4.2	3.7–6.5
	Mean fibrinogen (mg/dL)	600 (1/7)	NA	NA	100–400
Non-fatal EEHV-infected (EEHV DNAemia)^c^	No. of animals	8	4	NA	
	Mean age (year-old)	4.92	2.2	NA	
	Mean RBC count (× 10^6^ cells/µL)	3.09	2.63	NA	2.5–5
	Mean PCV (%)	35.37	30.25	NA	30–40
	Mean platelet count (× 10^3^ cells/µL)	316	355.75	NA	200–600
	Mean total serum protein (g/dL)	7.01 (6/8)	7.67	NA	6–8
	Albumin (g/dL)	2.84 (6/8)	2.95	NA	1.5–3.5
	Globulin (g/dL)	4.17 (6/8)	4.75	NA	3.7–6.5
	Mean fibrinogen (mg/dL)	466.66 (3/8)	NA	NA	100–400

*NA* not available, *RBC* red blood cells, *PCV* packed cell volume.

^a^Mikota^22^.

^b^Parenthesis indicates the number of calculated animals.

^c^Elephants that had been infected by EEHV but showed only mild non-fatal symptoms.

Blood monitoring of the EEHV1A-HD, EEHV4-HD and EEHV1A/4-HD cases indicate that a marked reduction in the platelet counts in elephant blood circulation could be an indicative factor of acute death since the platelet count in most of the fatal EEHV-HD cases dropped within 24 h prior the subject passing (Table 2).

**Table 2:** Platelet count of elephant calves prior to an EEHV-HD-related decease.

Animal ID	Age (year-old)	Platelet count (× 10^3^ cells/µL) on days prior to decease								
		Day 7	Day 6	Day 5	Day 4	Day 3	Day 2	Day 1	Day 0	Remark
1. HS/15	2	–	–	–	–	–	–	36	Deceased	EEHV1A
2. TT/18	2	–	–	–	–	254	147	41	Deceased	EEHV1A
3. MN/18	1	–	–	–	–	–	–	69	Deceased	EEHV1A
4. PW/19	3	–	–	–	131	–	–	74	Deceased	EEHV1A
5. NK/19	2	–	–	–	–	–	–	87	Deceased	EEHV1A
6. SB/20	7	–	–	–	–	–	–	56	Deceased	EEHV1A
7. SK/20	2.5	–	–	–	–	–	–	67	Deceased	EEHV1A
8. PP/18	4	–	–	–	–	–	–	101	Deceased	EEHV4
9. ML/17	2	355	–	–	–	–	–	40	Deceased	EEHV4
10. NN/17	5	–	–	–	–	–	–	173	Deceased	EEHV1A/4

### Histopathological analysis of vascular lesions in fatal EEHV-HD and EEHV-negative calves

**Figure 1:**
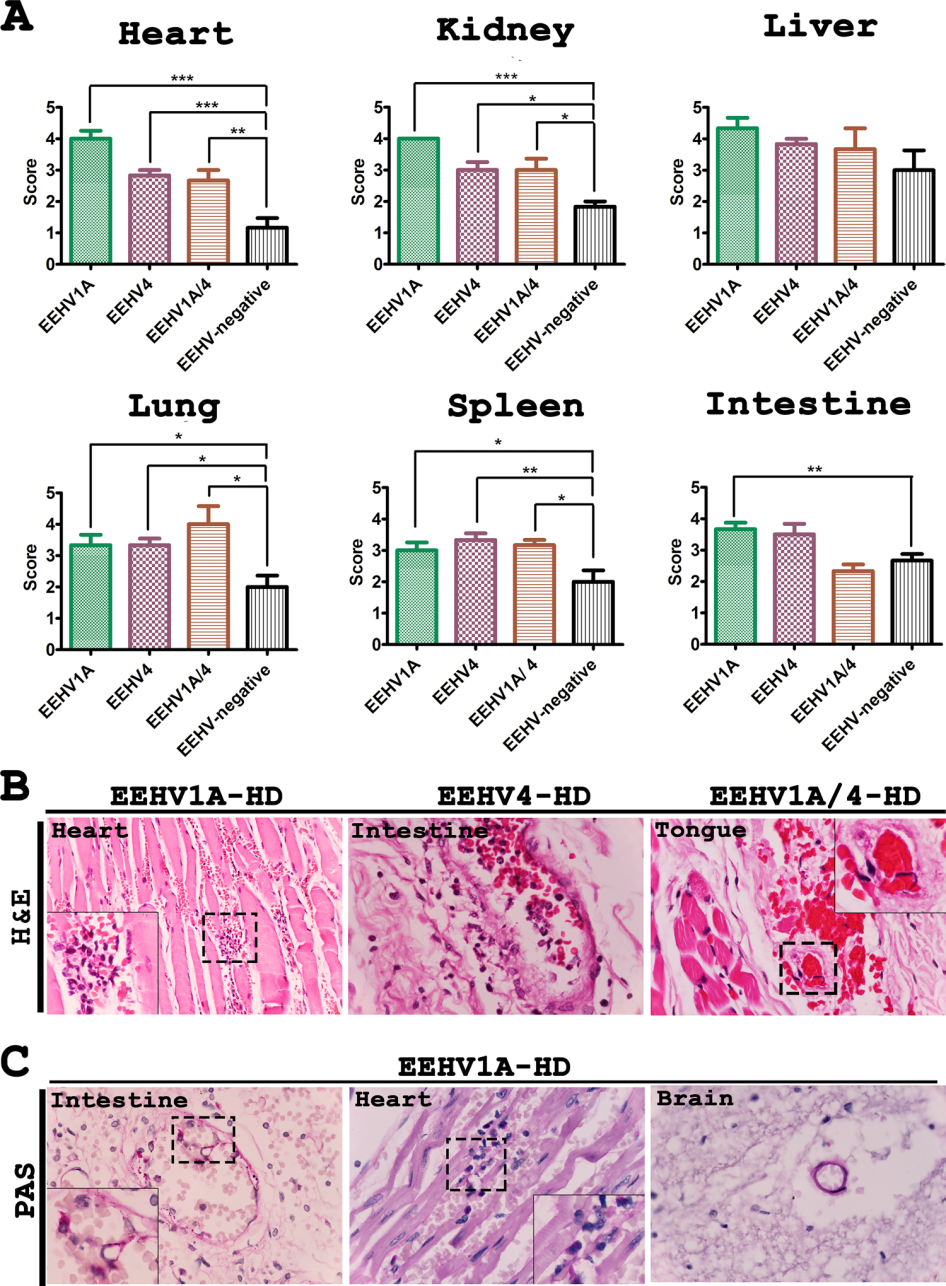
Grading of vascular lesions and representative histopathological photomicrographs of elephant calves that died due to EEHV-HD and unrelated EEHV infection. (**A**) EEHV1A-HD, EEHV4-HD or EEHV1A/4-HD were associated with a significantly greater amount of vascular lesions in the heart, kidneys, lungs and spleen when compared to the EEHV-negative controls, while infection with EEHV1A alone resulted in a significantly higher pathological score in the intestines. Furthermore, blood vessels in the liver displayed no remarkable pathological changes between the EEHV-HD cases and the EEHV-negative cases. Data are presented as mean ± standard error values. Asterisks indicate statistically significant differences (*p < 0.05, **p < 0.01, ***p < 0.001) when compared to the EEHV-negative controls. (**B**) H&E stain demonstrated lymphohistiocytic (EEHV1A, inset) or fibrino-necrotizing (EEHV4, inset) vasculitis and perivasculitis of the small blood vessels of the EEHV-HD calves. (**C**) PAS stain revealed disruption of the endothelial basal membrane of the internal organs, including the intestines and heart, of EEHV-HD calves (inset). Disruption of the vascular basement membrane was not observed throughout the central nervous system such as in the brain.

The grading of vascular lesions in elephant tissues using H&E stain indicated that the heart, kidneys, lungs, spleen and intestines, of deceased EEHV-HD calves had a significantly greater amount of severe vascular lesions when compared to the EEHV-negative controls (Fig. 1A). The vascular lesion scores in the EEHV-HD cases were correlated with the high viral genome copies that were found in various internal organs including the heart, kidneys, lungs, spleen and intestines (Table S2). However, despite the fact that EEHV genomes were detected at a high value, the vascular lesions in the liver were not found to be significantly different (Fig. 1A and Table S2). It has been noted that blood vessels that show significant pathological lesions were typically limited to small arteries, arterioles, capillaries and venules (Fig. 1B). In this case, these histopathological lesions ranged from mild to severe in terms of the fibrinonecrotizing or lymphohistiocytic arteritis or lymphangitis/phlebitis with various degrees of hemorrhaging (Fig. S1). Perivascular tissues were also found to have expanded in relation to the edema and infiltrating inflammatory cells (Fig. S1). The periodic acid-Schiff (PAS) stain revealed disruption of the endothelia and basement membrane of the EEHV-HD small blood vessels of the visceral organs (Fig. 1C). However, blood vessels in the central nervous system (CNS), including the brain and spinal cord, were found to be unaltered when compared to the EEHV-negative controls (Fig. 1C). Medium and large blood vessels were observed to show a limited degree of inflammation in the vasa vasorum of the tunica adventitia and media (Fig. S1). These findings suggest that small blood vessels, rather than large blood vessels, are damaged during acute fatal EEHV-HD.

### Thromboemboli in blood vessels of fatal EEHV-HD cases

**Figure 2:**
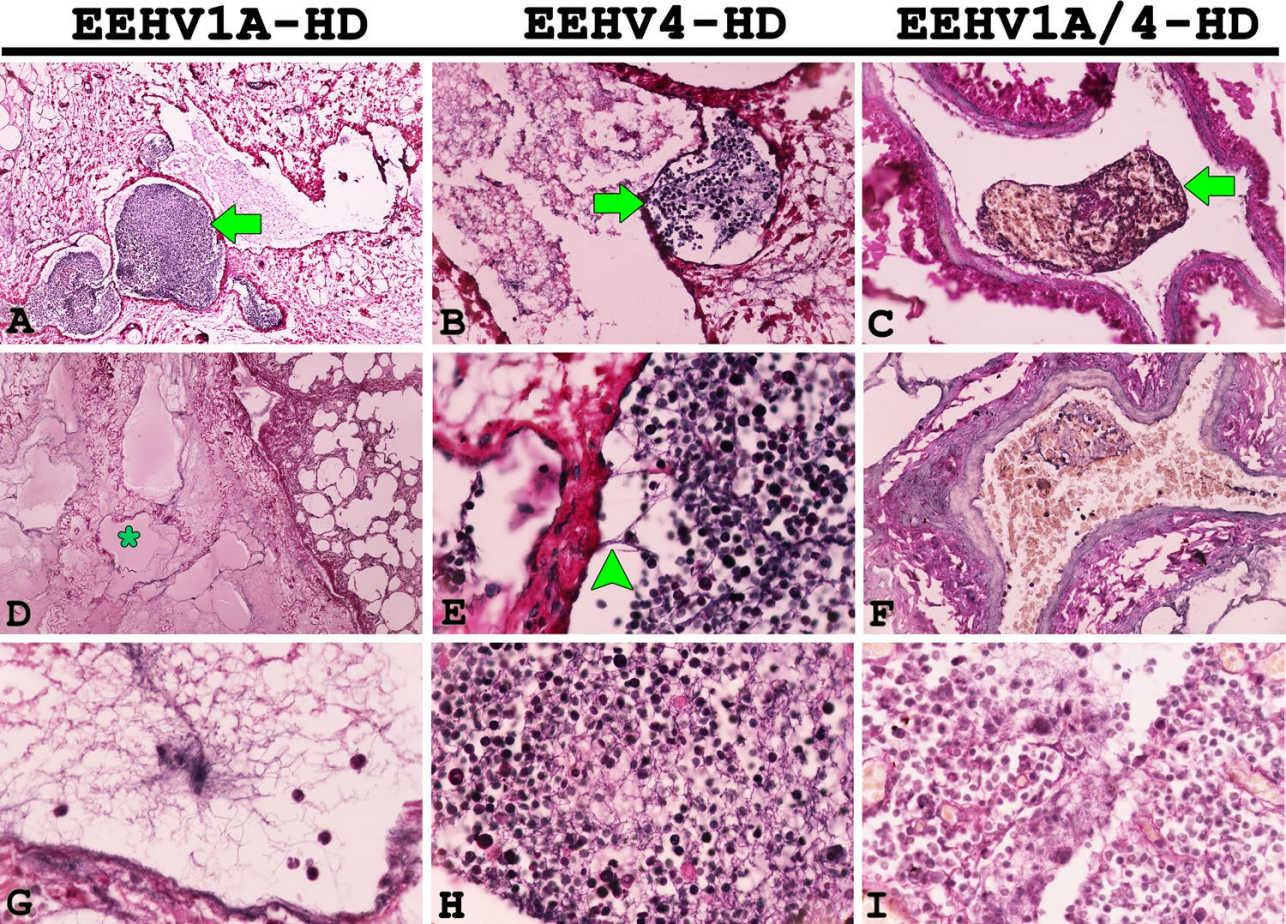
Representative photomicrographs of phosphotungstic acid hematoxylin (PTAH) stains of EEHV-HD and EEHV-negative cases. PTAH-positive fibrin formation was seen in the blood vessels and extravascular spaces of EEHV1A-HD (**A**, **D**, **G**), EEHV4-HD (**B**, **E**, **H**), and EEHV1A/4-HD (**C**, **F**, **I**) calves. A PTAH-positive result was indicated by a deep blue to black color fibrin formation of the thrombi/emboli within the lumen of blood vessels (arrow), as well as in the extracellular space (star, **D**) or by adhering to the vessel wall (arrowhead, **E**).

Phosphotungstic acid hematoxylin (PTAH) stain demonstrated that fibrin formation was predominantly observed as thromboemboli in the blood vessels or extravascular space of the internal organs including the lungs, heart, kidneys and intestines of EEHV1A-HD, EEHV4-HD and EEHV1A/4-HD calves (Fig. 2).

### Mast cells are less likely to increase vascular permeability in acute fatal EEHV-HD

**Figure 3:**
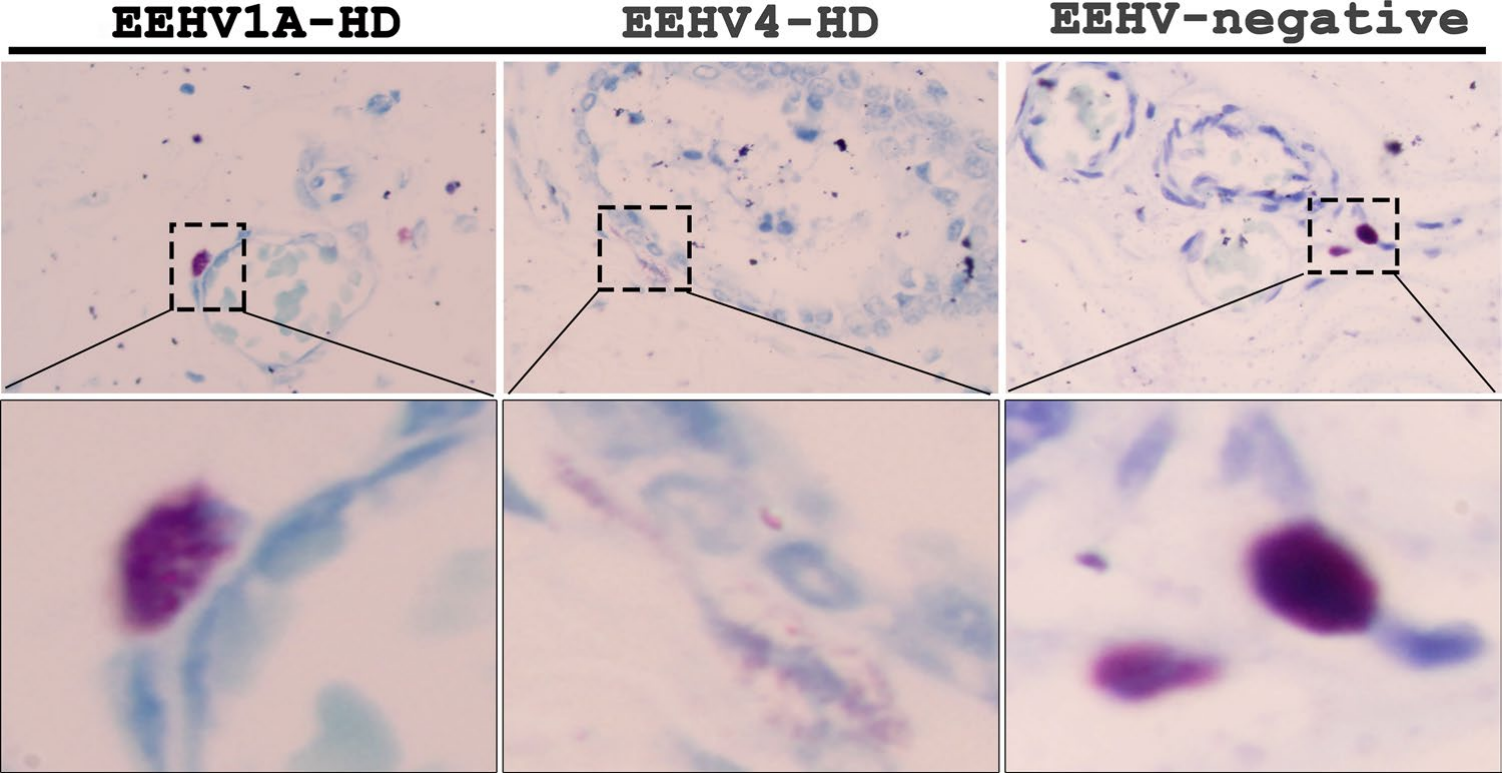
Representative photomicrographs of toluidine blue stain of EEHV-HD and EEHV-negative cases. Non-degranulated mast cells that appeared as variable degrees of moderated to large purple or metachromatic granules in the cytoplasm (inset) were observed around the blood vessels of the EEHV-HD and EEHV-negative subjects. The number of mast cells around the blood vessels were found to not be significantly different in comparisons that were made among the EEHV1A-HD, EEHV4-HD and EEHV-negative control animals.

Since degranulation of mast cells plays a central role in the increase of vascular permeability in several diseases caused by viruses^21, 23, 24^, this study investigated whether this mechanism typically occurs in EEHV-HD cases. Our results indicate that neither proliferation nor degranulation of perivascular mast cells was different in comparisons made between fatal EEHV-HD cases and the EEHV-negative controls (Fig. 3).

### Increase of PECAM-1-immunolabeling positive cells in EEHV-HD cases

**Figure 4:**
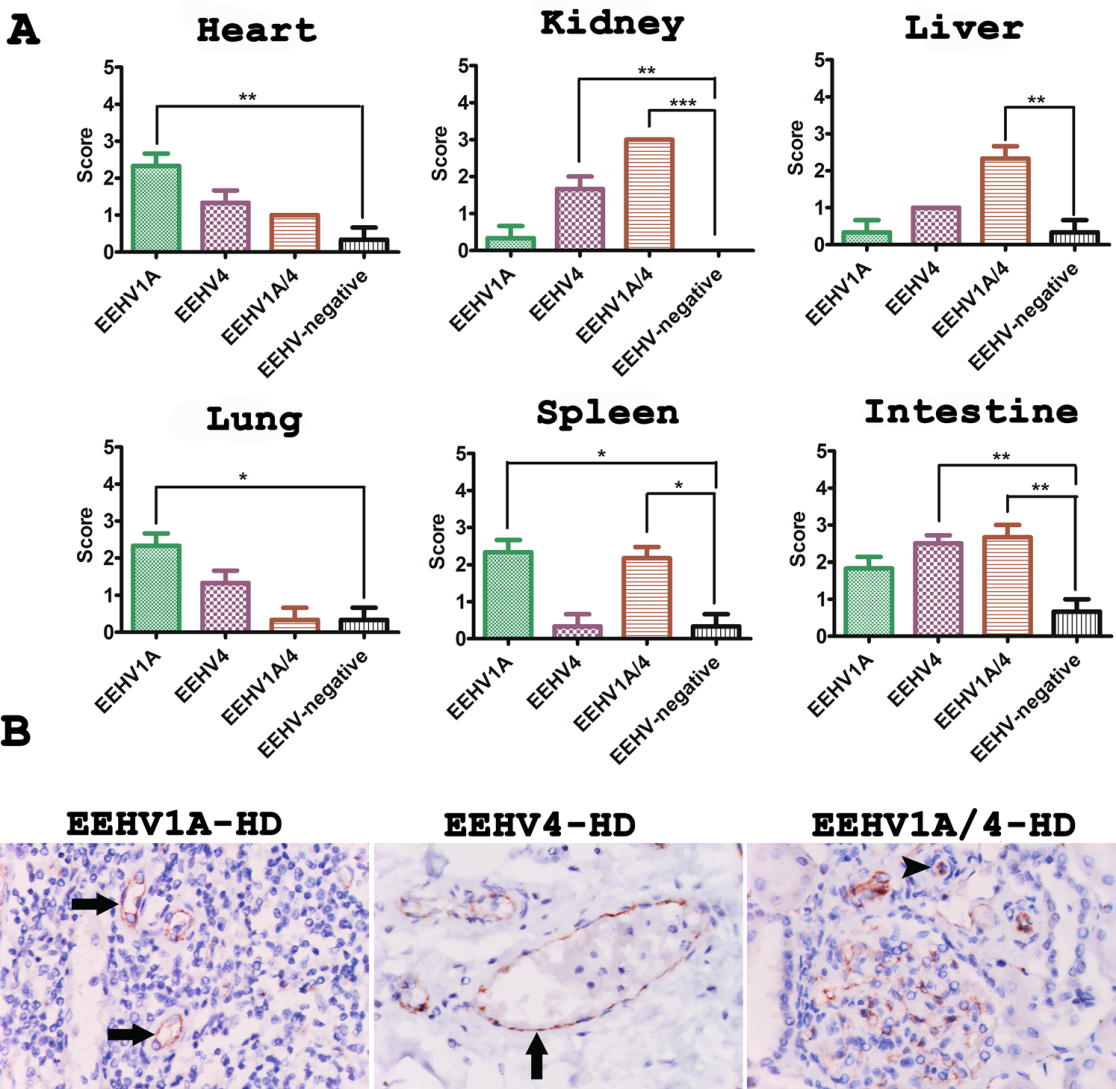
Scoring and representative photomicrographs of PECAM-1 positive cells in EEHV-HD and EEHV-negative tissues. (**A**) PECAM-1 immunolabeling positive cells were significantly observed in the internal organs of EEHV-HD subjects when compared to the EEHV-negative controls. PECAM-1 expression was noticeable in the heart, lungs, and spleen of EEHV1A-HD subjects, while it was significantly observed in the kidneys and intestines of the EEHV4-HD subjects and in the kidneys, liver, spleen and intestines of EEHV1A/4-HD subjects. Asterisks indicate statistically significant differences (*p < 0.05, **p < 0.01, ***p < 0.001) when compared to the EEHV-negative controls. (**B**) Immunolabeling positive cells for PECAM-1 included the endothelia (arrow) and leukocytes (arrowhead) that adhered to the vessel wall.

Since PECAM-1 expression was determined to be a marker for activated endothelia and could be used to determine adhesion of platelets or leukocytes to damaged endothelia^25^, immunohistochemistry (IHC) for PECAM-1 was performed in this study. The results indicated that PECAM-1 positive cells were shown to be significantly observed in EEHV-HD cases when compared to the EEHV-negative controls (Fig. 4). However, it should be noted that the degree of PECAM-1 expression was found to be different in different organs of EEHV1A-HD, EEHV4-HD and EEHV1A/4-HD cases (Fig. 4A).

### Increase of von Willebrand factor (vWF)-immunolabeling positive cells in EEHV-HD cases

**Figure 5:**
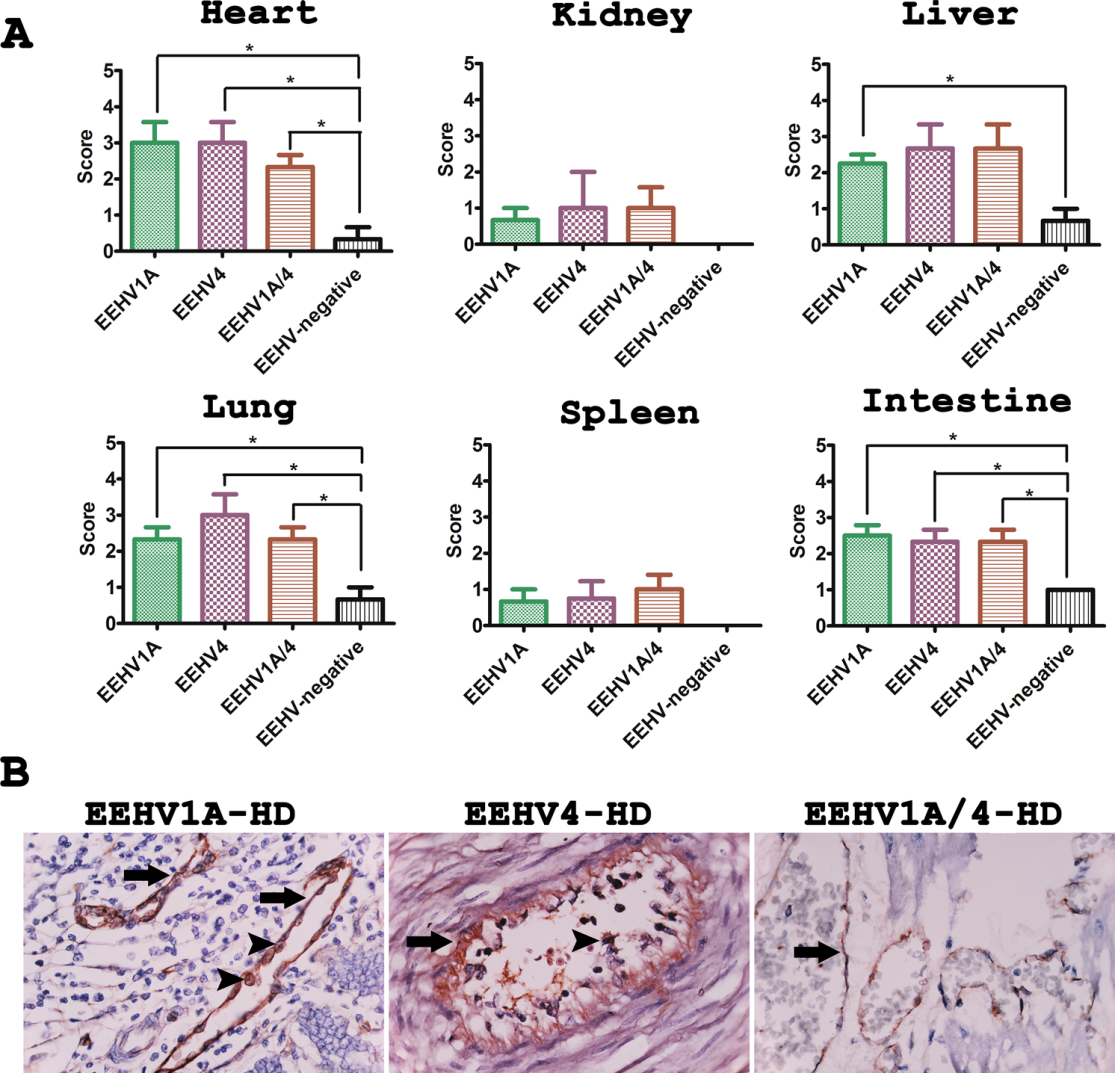
Scoring and representative photomicrographs of von Willebrand factor (vWF) immunolabeling positive cells in EEHV-HD and EEHV-negative calves. (**A**) Immunolabeling of vWF was shown to be significantly expressed in the heart, lungs and intestines of EEHV1A-HD, EEHV4-HD and EEHV1A/4-HD calves, while only EEHV1A-HD revealed a significant degree of expression of vWF in the liver when compared to the EEHV-negative controls. Notably, expression of vWF did not differ in the kidneys and spleen in comparisons made between EEHV-HD and EEHV-negative cases. Asterisks indicate statistically significant differences (*p < 0.05). (**B**) The vWF immunolabeling positive cells were observed in the endothelia (arrows) of the blood vessels and leukocytes (arrowheads) that were tethered to the endothelia.

IHC for the detection of vWF antigens revealed that positive cells were significantly observed in the heart, lungs and intestines of the EEHV1A-HD and EEHV4-HD cases, while the expression of vWF in the kidneys, spleen and liver was found to be non-significantly different when compared to the EEHV-negative controls (Fig. 5A). Importantly, not only was vascular endothelia expressed in the vWF antigens, but leukocytes that adhered to the endothelia were also stained by the vWF antibody (Fig. 5B).

### Up-regulation of cytokine mRNA in tissue samples of EEHV-HD calves

**Figure 6:**
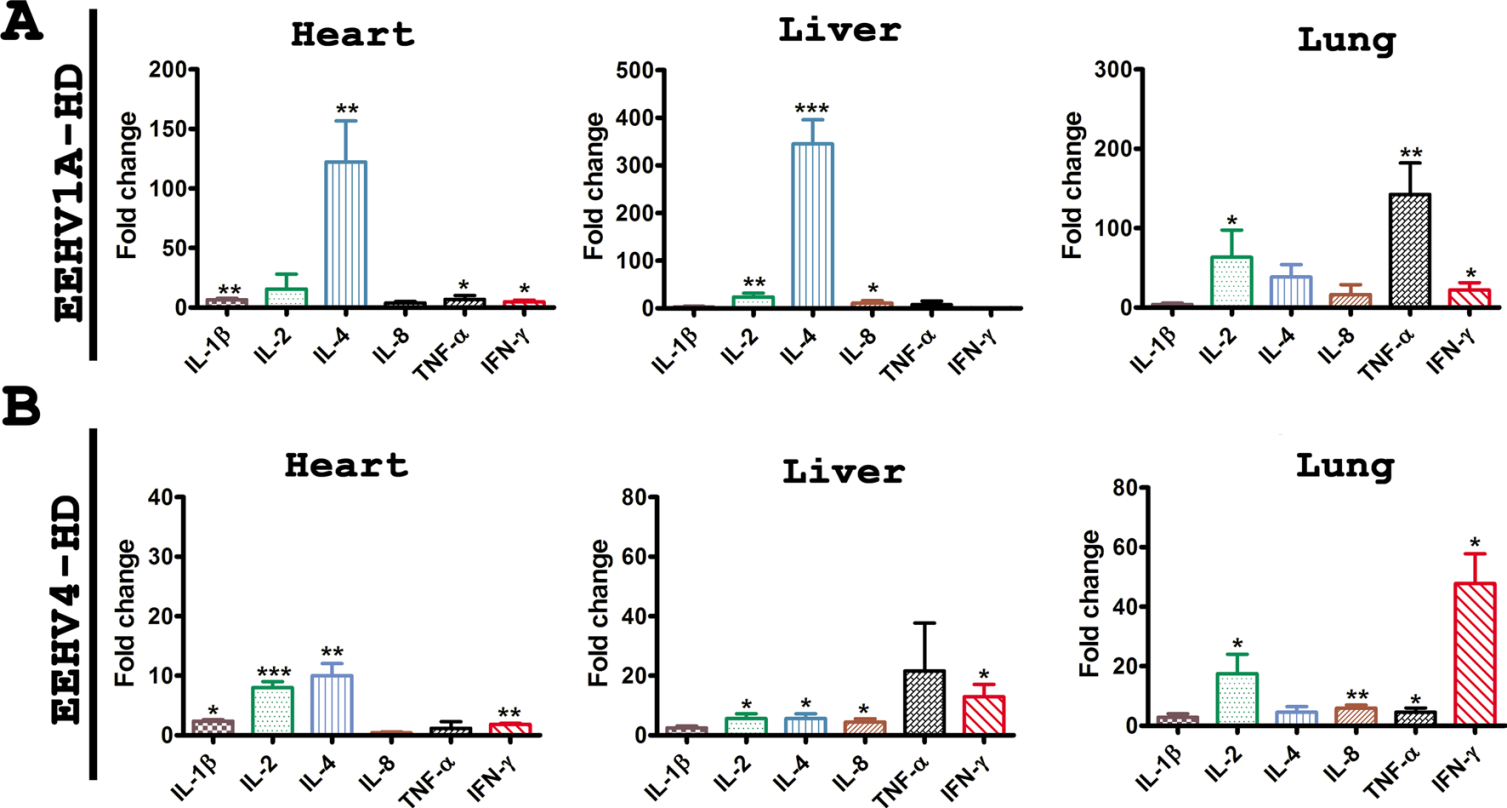
Cytokine mRNA expression in the tissues of acute EEHV-HD cases. Expressions of pro-inflammatory and inflammatory cytokines, including IL-1β, IL-2, IL-4, IL-8, TNF-α and IFN-γ, were significantly observed in the heart, liver or lungs of the EEHV-HD cases when compared to the EEHV-negative controls. Data are presented as the mean ± standard error values from two independent experiments. Asterisks indicate statistically significant differences (*p < 0.05, **p < 0.01, ***p < 0.001) when compared to the EEHV-negative controls using GAPDH as an internal control.

Quantification of cytokine mRNA expression in EEHV1A-HD tissues indicated a significant degree of up-regulation of IL-1β, IL-4, TNF-α and IFN-γ expression in the heart, while the liver and lungs showed a significant degree in mRNA up-regulation of IL-2, IL-4, IL-8 and IL-2, TNF-α and IFN-γ expression, respectively (Fig. 6). Meanwhile, samples of the heart, liver and lungs of the EEHV4-HD cases also revealed a significant degree of up-regulation of several cytokines, including IL-1β, IL-2, IL-4, IL-8, TNF-α and IFN-ɣ, when compared to the un-related EEHV-infected cases (Fig. 6).

## Discussion

**Figure 7:**
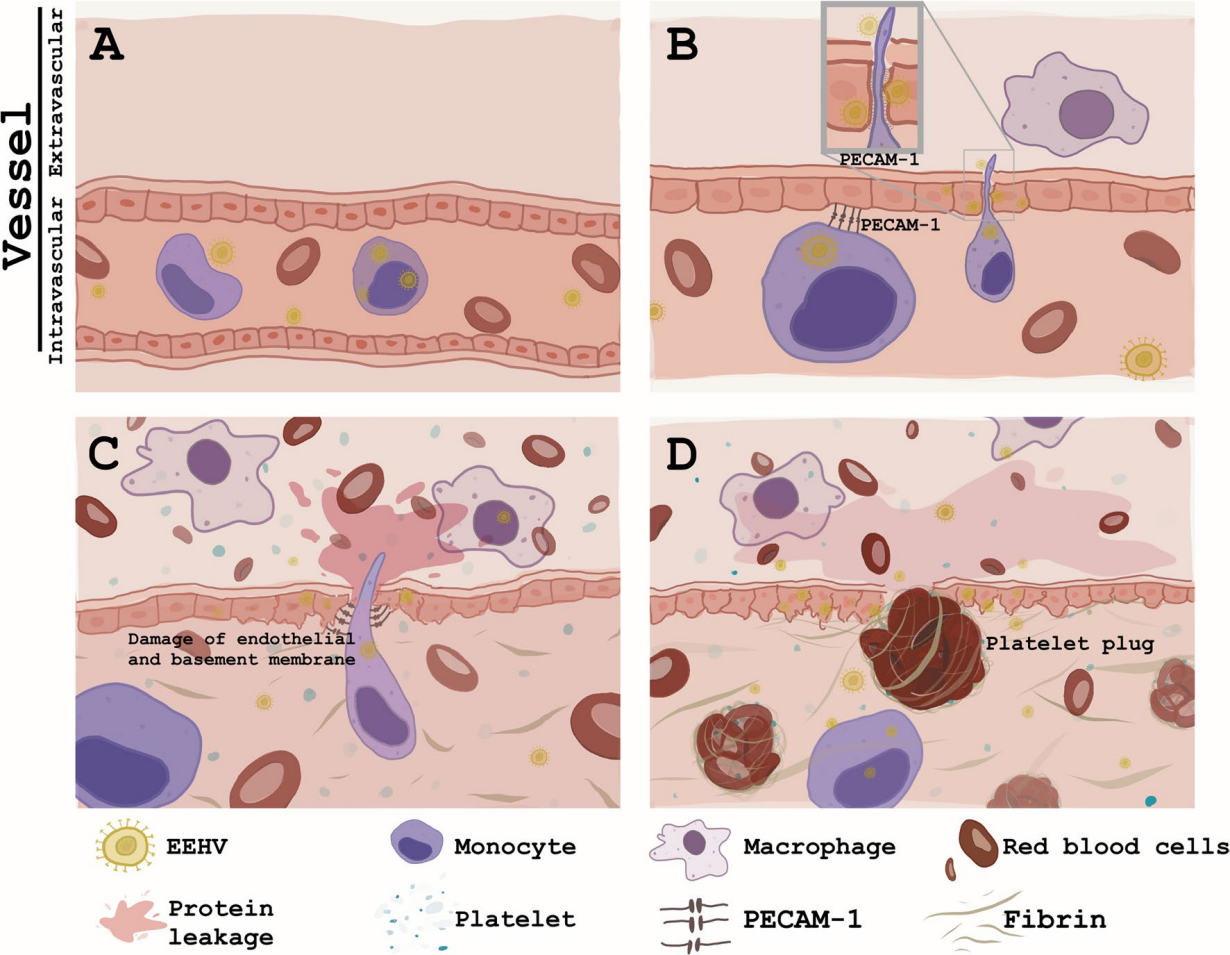
Proposed schematic mechanisms of hemorrhagic and edematous lesions in acute EEHV-HD in Asian elephants. During the viremic phase, EEHV was transmitted from virus-infected monocytes (**A**) to the endothelia by adhesion of monocytes to specific molecules such as PECAM-1 (**B**). After infection and replication occurred in the endothelia, EEHV induced endothelial damage throughout the body and caused diffused hemorrhaging and edema of the internal organs (**C**). Subsequently, together with the up-regulation of inflammatory cytokines by virus-infected cells, EEHV-infection also altered the coagulation system and caused thrombo-emboli in the blood vessels, which then enhanced hemorrhagic and edematous lesions in the EEHV-HD cases (**D**).

In this study, we have demonstrated and proposed that massive endothelial destruction and systemic inflammation caused by EEHV dysregulated blood coagulation systems resulting in severe hemorrhaging and edema. EEHV in elephants is disseminated throughout the body via the circulation of EEHV-infected blood monocytes^6^. In small blood vessels or microvessels, EEHV was transmitted from virus-infected monocytes to the endothelia by adhesion of monocytes to specific molecules present in the endothelia. This would then allow the endothelia to be infected and to serve as replication sites for EEHV^7^. After infection and replication occurred in the endothelia, EEHV induced endothelial damage throughout the body and caused diffused hemorrhaging and edema of the internal organs. Subsequently, together with the up-regulation of inflammatory cytokines (so-called “cytokine storm”) by virus-infected cells, EEHV-infection also altered the coagulation system and caused thrombo-emboli in the blood vessels. These complex processes lead to disseminated intravascular coagulopathy, which could further enhance bleeding tendencies in EEHV-HD cases (Fig. 7). Consequently, elephants may die due to hypovolemic shock and/or organ failure. These processes may be initiated and end as rapidly as 24 h after the onset of the first clinical signs of EEHV-HD. Depending upon the EEHV genotypes, the degree of thrombocytopenia, the affected organs, the severity of the vascular lesions and the viral loads differed among EEHV-infected Asian elephants. Nevertheless, most elephants that succumbed and died due to EEHV-HD presented significant hematological signs of thrombocytopenia and systemic coagulative disorder.

Activation of coagulation cascades during inflammation is the result of the stimulation of coagulation synthesis, a decrease in the synthesis of anti-coagulants and the suppression of fibrinolysis^26^. These complex cascades are initiated when vascular endothelial damage occurs and there is exposure of subendothelial collagen to von Willebrand factors (vWF), a multimeric glycoprotein present in blood plasma, the subendothelial matrix, endothelial cells and platelets^11, 27, 28^. The binding of vWF to the subendothelial matrix then mediates the adhesion and aggregation of platelets at sites of vascular injury. This is followed by fibrin polymerization and the formation of platelet plugs to cease blood loss^29, 30^. Since the roles of vascular damage and coagulation have not yet been investigated in EEHV-HD cases, our findings demonstrate that disruption of endothelia resulted in increased expressions of vWF and PECAM-1. The pathomechanisms of hemorrhaging and edematous lesions in EEHV-HD cases were consistent with the outcomes observed for other virus-induced hemorrhagic diseases^14^. Moreover, our findings also suggest that the up-regulation of pro-inflammatory and inflammatory cytokines, including IL-1β, IL-2, IL-4 and TNF-α, may also be involved in the pathogenesis of acute hemorrhagic diseases as can be seen in the influenza virus or dengue hemorrhagic fever^31, 32^. Excessive immune/inflammatory reactions (cytokine storm) have been shown to exacerbate the coagulopathy in many viral-infectious diseases^32–36^. Thus, it can be presumably concluded that diffused endothelial damage and platelet activation result in platelet plugs within the injured vessels or intravascular network. This can lead to thrombocytopenia which is known as disseminated intravascular coagulation (DIC) in the acute hemorrhagic disease caused by EEHV infection^37, 38^.

DIC is a complex process, that is characterized by abnormal thrombin generation. This can then lead to diffused fibrin formation, especially within microcirculation and thereby the consumption of platelets, fibrinogen, coagulation factors and inhibitors^13, 39^. DIC plays a major role in the hemorrhaging and edema that occur in several viral hemorrhagic diseases in humans and animals such as the Ebola virus, avian influenza, African swine fever, Marburg disease or severe acute respiratory syndrome (SARS)^17, 38, 40, 41^. Despite the fact that there is no gold standard for the diagnosis of DIC and no specific biomarkers that can be employed to clearly diagnose DIC in humans^42^, clinical laboratory diagnosis of DIC in animals can be accomplished when three or more of the following abnormalities are found; (1) the prothrombin time (PT) is 3 or more seconds greater than the control or partial thromboplastin time (PTT) is 5 or more seconds greater than the upper limit of normal; (2) the presence of elevated D-dimer; (3) a decrease in platelet count; (4) an absolute decrease in plasma fibrinogen levels ≥ 25%; and (5) the presence of positive PTAH staining for intravascular fibrin formation^43, 44^. Due to limitations in the availability of blood samples, PT and PTT tests of EEHV-HD cases were not conducted. Moreover, the plasma fibrinogen count of the acute EEHV-HD cases observed in this study was not significantly different when compared to the EEHV negative cases. However, this outcome could have been due to a severe loss in fluid and protein that occurred from blood circulation. Fatal EEHV-HD cases may result in a total reduction in blood cells and plasma proteins in relation to the fluid volume obtained from blood vessels. The massive fluid loss from the blood vessels may then influence the absolute fibrinogen count in the blood of elephants. Thus, we have suggested that red blood cell counts, PCV or total plasma proteins may not be appropriate hematological parameters for determining the fatality signs of acute EEHV-HD. However, the fact that a reduction in the platelet count, as well as the presence of PTAH positive fibrin thrombi in the blood vessels of EEHV-HD calves in the present study, strongly suggest that DIC occurs as a result of, and is directly involved in, pathogenesis of a bleeding disorder in EEHV-HD calves.

We have hypothesized that thrombocytopenia in EEHV-HD cases is less likely to be caused by platelet destruction or a reduced level of production of the platelets. This conclusion can be explained by the fact that the average platelet count in non-fatal EEHV-infected calves was determined to be within a normal reference value when compared to the EEHV-negative controls. This conclusion was further emphasized by findings that a sudden fall in the platelet count occurred within 24 h prior to the acute death of elephant calves that could have been due to utilization of platelets during primary hemostasis^42^. In addition, the H&E staining of elephant bone marrow samples in our previous study indicated that there were no differences in the relative number of megakaryocytes in the EEHV-HD and EEHV-negative cases^7^. The data suggest that thrombocytopenia in EEHV-HD calves is less likely due to a reduction in platelet production. On the other hand, acute onset of thrombocytopenia suggests that it is more likely due to the consumption rather than the destruction of platelets in fatal EEHV-HD cases. More specifically, platelet destruction is found to be predominantly observed in the immune-mediated thrombocytopenia such as drug-induced immune mediated thrombocytopenia and autoimmune diseases^45–48^. Collectively and based on this conclusion, therapeutic intervention of acute EEHV-HD cases through the use platelet-rich plasma (PRP) is highly recommended.

The findings of our study suggest that plasma leakage in the EEHV-HD elephant cases was not due to mast cell activation and degranulation. An increase in the degree of plasma leakage from blood circulation is one of the most striking outcomes of several viral hemorrhagic diseases such as Dengue hemorrhagic fever^19–21^. It has been shown that degranulation of mast cells can increase vascular permeability and cause vascular leakage in Dengue hemorrhagic fever patients^19–21, 49^. These effects were evidenced by a relatively low degree or absence of tissue inflammation, endothelial cell edema and perivascular edema^50^. Activation of mast cells around the blood vessels was observed by an increase in the number and release of chromogenic granules, which can be detected by the toluidine blue stain. The fact that only small amounts of granulated and degranulated mast cells were observed around the medium and small blood vessels of EEHV-HD calves suggests that vascular hyperpermeability in these cases was unlikely caused by the activation of mast cells. On the other hand, it should be noted that the up-regulation of cytokines and inflammatory mediators during virus infection can also cause endothelial contraction and could enhance vascular permeability in in vitro and in vivo experiments^51–54^. It remains to be determined if the up-regulation of IL-1β, IL-2, IL-4, IL-8 and TNF-α observed in this study was involved in vascular hyperpermeability. Notably, other tissue factors, such as those associated with nitric oxide, are also known to be cellular mediators that can increase vascular permeability^55^. Therefore, the role of other cellular mediators in EEHV infection needs to be further investigated.

In conclusion, our present study suggests that DIC did occur in EEHV-HD cases among Asian elephants. Furthermore, DIC together with a disruption of small blood vessels can lead to diffused hemorrhaging of the internal organs. Diffused, severe hemorrhaging and edema of the subcutaneous tissue and internal organs may lead to hypovolemic shock and ultimately lead to fatalities in EEHV-HD calves. The present study also demonstrated that, unlike Dengue hemorrhagic fever, mast cell degranulation is less likely a cause of vascular and protein leakage in EEHV-HD cases. Thus, based on our current understanding of the EEHV pathogenesis, treatment of EEHV-HD cases should be included antiviral drugs, cytokine storm treatment and antithrombotic therapy. Moreover, application of antioxidants, such as vitamin C, is also suggested as a prophylactic support against EEHV-HD. To summarize, the present study has brought attention to the pathogenesis of EEHV coagulopathy and may be useful for further therapeutic intervention in EEHV-infected Asian elephants.

## Methods

### Samples and tissue processing

The profiles of blood analyses that can intensely influence the vascular coagulation system, specifically in the manner of platelet count and plasma protein concentrations, and archived of formalin-fixed, paraffin-embedded (FFPE) tissues or frozen tissues of fatal EEHV-HD, non-fatal EEHV-infected and EEHV-negative cases were obtained from the Veterinary Diagnostic Center, Faculty of Veterinary Medicine, Chiang Mai University, Thailand between the years of 2015 and 2020 (Table 1 and Table S1). The blood of these elephants was obtained at either routine annual health checkups or clinical routine monitoring during the course of EEHV infection. The EEHV-negative samples used in this study were taken from a calf that had been diagnosed as stillbirth and a calf that died due to un-related EEHV infection^6, 7^. In cases of non-fatal EEHV-infection or fatal EEHV-HD, subjects were further diagnosed to specify the EEHV genotypes using PCR and gene sequencing, as has been previously described^4^. FFPE samples of EEHV1A-HD (n = 3), EEHV4 (n = 2), EEHV1A/4-HD (n = 1) and EEHV-negative calves (n = 2) were 3 µm-thick cut and processed for further histopathological investigation by being stained with hematoxylin and eosin (H&E) stain^4^, periodic acid-Schiff (PAS) stain, toluidine blue stain or Mallory's phosphotungstic acid hematoxylin (PTAH) stain. This entire process will be described in greater detail below. Samples subjected to immunohistochemistry were 3 µm-thick and obtained on 3-Aminopropyl-triethoxysilane coated-slides. In addition, frozen tissue samples of the EEHV1A-HD, EEHV4-HD, EEHV1A/4-HD and EEHV-negative cases were subjected to further analysis in order to determine viral loads and the degree of cytokine mRNA expression using quantitative PCR and RT-PCR, as has been previously described^6, 56, 57^. All experimental animal protocols in this study were consistent with the Guide for the Care and Use of Laboratory Animals (National Institute of Animal Health, WA). The blood sampling protocol that was used for animals in this study was approved of by the Faculty of Veterinary Medicine, Chiang Mai University Animal Care and Use Committee (License number S22/2563).

### Periodic acid-Schiff (PAS) staining

To investigate the basement membrane of elephant blood vessels, periodic acid-Schiff (PAS) staining was done according to the method previously described^58^. Briefly, 3 µm-thick sections were deparaffinized and rehydrated. They were then incubated with a periodic acid solution for 5 min at room temperature (RT). After being washed with tap water, slides were then incubated in Schiff solution for 15 min at RT and then washed again with tap water. Nuclei were visualized with hematoxylin prior to being dehydrated and mounted with a cover glass. The slides were observed and photos were taken using a light microscope.

### Toluidine blue staining

To identify mast cells in elephant tissues, toluidine blue stain was used. Briefly, after deparaffinization and rehydration, sections were stained with 0.1% toluidine blue for approximately 15 s and then quickly washed three times with distilled water for 5 min each. They were then incubated with 30% alcohol and dehydrated by sequentially dipping them for approximately 5 s each in 75%, 85%, 95% and 100% alcohol, respectively. Thereafter, sections were dipped in xylene for 5 min, mounted with cover glasses and examined under a light microscope. Intact mast cells showed deep magenta staining, while degranulated mast cells showed purple or magenta granules that extruded adjacent to the mast cells. The intact and degranulated mast cell numbers in each slide (organ) were randomly counted from 10 high power fields, as has been previously described^59^.

### Phosphotungstic acid hematoxylin (PTAH) stain

To examine the fibrin formation within and outside the blood vessels, phosphotungstic acid hematoxylin (PTAH Stain Kit, Abcam, Cambridge, UK) staining was done according to the manufacturer’s instructions. Briefly, FFPE slides were deparaffinized and rehydrated with xylene and a series of graded alcohol solutions. Slides were then incubated with agitation in a warmed zinc chloride solution for 15 min. After being washed with running tap water, slides were further incubated in warmed ferric ammonium sulphate aqueous solution for 2 min. Thereafter, they were rinsed with running tap water for 2 min and incubated with a warmed PTAH solution for 30 min. Differentiations in color were observed in 95% alcohol until the desired stain was obtained. Slides were then dehydrated, mounted with permount and coverslips, and then observed under a light microscope.

### Grading of vascular lesions

Grading of the vascular lesions was done by three independent pathologists who were not aware of the sample groups. Vascular lesions were categorized, according to the method previously described^60^ with slight modifications, where grade 0 = normal finding, grade 1 = mild dilation of small blood vessels with no alteration of blood vessel walls, grade 2 = increased blood volume and strong vasodilation with erythrocyte accumulation, grade 3 = transmural rupture of a small number (up to 50%) of blood vessels with accumulation of the inflammatory cells, grade 4 = complete loss of the basement membrane and endothelial cells of a large number (> 50%) of blood vessels and grade 5 = diffused hemorrhaging of the tissues with or without visible blood vessels. The areas assessed included the endothelia of the capillaries, large, medium and small blood vessels of the heart, kidneys, liver, lungs, spleen, and the small and large intestines of fatal EEHV-HD cases. The results of the analysis were then compared to the EEHV-negative controls.

### Immunohistochemistry

Immunohistochemistry was performed by employing the avidin–biotin complex (ABC) method as has been described previously^6, 61^. Briefly, FFPE sections were deparaffinized, rehydrated and then microwaved for 30 min in citrate buffer (pH 6.0). Sections were incubated for 5 min with 3%H_2_O_2_ in methanol and then blocked for 1 h at RT with PBS containing 5% normal goat serum and 0.1% Triton X-100. Thereafter, they were incubated for 2 h at RT with primary antibodies. The primary antibodies used in this study included the rabbit anti-platelet endothelial cell adhesion molecules-1 (PECAM-1; 1:400) and rabbit anti-von Willebrand factor (vWF; 1:400, all obtained from Abcam, Cambridge, UK). After being washed, sections were incubated for 45 min at RT with biotinylated secondary antibody (1:200; Vector Laboratories, CA, USA), followed by peroxidise coupled ABC (Thermo Fischer Scientific, Waltham, MA, USA). Antibody binding was visualized using DAB-H_2_O_2_ for 5 min at RT followed by being counterstained with hematoxylin. They were then dehydrated, mounted with coverslips and observed under a light microscope. Scoring of immunolabeling positive cells for each antibody in different anatomical regions was done according to the method previously described^6^.

### Quantitative PCR and RT-PCR

To quantify the viral genome copies (vgc) of EEHV in various organs, total DNA of frozen EEHV1A-HD, EEHV4-HD and EEHV1A/4-HD tissues (heart, lungs, liver, spleen, kidney and intestines) were extracted using a commercial DNA extraction kit (NucleoSpin Tissue, Machery-Nagel GmbH, Düren, Germany). Primers of the terminase gene for the identification of EEHV1 and the polymerase gene for the identification of EEHV3/4 were used according to the previous studies^3, 57^. For the calculation of the number of viral copies, the target genes were amplified from total viral DNA. Conventional PCR cycling was initiated at 98 °C for 1 min followed by 98 °C for 10 s, 50 °C for 20 s, 72 °C for 15 s (35 cycles), and a final extension at 72 °C for 7 min using Phusion High-Fidelity PCR Master Mix (Thermo Scientific, USA). PCR products were then cut and purified from agarose gel using a GeneJET Gel Extraction and DNA Cleanup Micro Kit (Thermo Scientific, USA). The dsDNA concentrations were quantified from UV/Vis spectrophotometer (DU730; Beckman Coulter, USA) and viral copy numbers were determined and calculated as has been previously described^56^. DNA was resuspended in water and were 10-fold diluted from 10^10^ to 10^0^ copies in number. Absolute quantification in qPCR was performed under the following conditions: 95 °C for 2 min, 40 cycles of 95 °C for 5 s, 50 °C for 30 s, 72 °C for 30 s, and a final extension step at 72 °C for 10 min using Sensifast SYBR-Hi Rox (Bioline, Luckenwalde, Germany) with a ABI7300 thermocycler (Applied Biosystems, CA, USA). The differences between the threshold cycles (Ct) and Log value of the copy numbers were used to simulate linear regression, which was then used to estimate the viral copy numbers in 100 ng of DNA in each organ.

In order to quantify the expression of pro-inflammatory and inflammatory cytokines between the fatal EEHV-HD and the EEHV-negative control calves, real time PCR (SYBR green selected master mix, Life Technologies, CA, USA) was performed in a total reaction volume of 20 μL. Primers of IL-1β, IL-2, IL-4, IL-8, TNF-α, IFN-γ and GAPDH were used as has been previously described^6^. PCR conditions were 95 °C for 10 min, followed by 40 cycles of 95 °C for 15 s, 60 °C for 30 s, and 72 °C for 30 s. The Ct of all genes were used to calculate the degree of gene expression using 2^−∆∆CT^ method^62^ normalized to that of the GAPDH gene and compared to the EEHV-negative controls, as has been previously described^6^.

### Statistical analysis

Statistical analyses were conducted using GraphPad Prism 5 (GraphPad Inc., La Jolla, CA, USA). Either unpaired t-test or one-way analysis of variance (ANOVA) was performed using mean or median values, followed by Tukey’s *post hoc* test depending upon the data types. Statistical significance was designated at *p* ≤ 0.05.

## Supplementary Information

Supplementary Information.
