# Supplementary material for: Pathogenesis of hemorrhagic disease caused by elephant endotheliotropic herpesvirus (EEHV) in Asian elephants (Elephas maximus)
Source: Sci Rep. 2021 Jun 21;11:12998. doi: 10.1038/s41598-021-92393-8 (PMC8217522; doi:10.1038/s41598-021-92393-8)
Supplement: Supplementary file 1 — Supplementary Information. [file 41598_2021_92393_MOESM1_ESM.pdf]

Pathogenesis of hemorrhagic disease caused by elephant endotheliotropic herpesvirus (EEHV) in Asian elephants (*Elephas maximus*)

Thunyamas Guntawang[1¶], Tidaratt Sittisak[1¶], Varankpicha Kochagul[2], Saralee Srivorakul[2], Kornravee Photichai[2], Kittikorn Boonsri[2], Thittaya Janyamethakul[3], Khajohnpat Boonprasert[4], Warangkhana Langkaphin[5], Chatchote Thitaram[4,6], Kidsadagon Pringproa[1,4*]

[1] Department of Veterinary Biosciences and Veterinary Public Health, Faculty of Veterinary Medicine, Chiang Mai University, Chiang Mai 50100, Thailand

[2] Veterinary Diagnostic Laboratory, Faculty of Veterinary Medicine, Chiang Mai University, Chiang Mai 50100, Thailand

[3] Patara Elephant Farm, Hang Dong, Chiang Mai 50230, Thailand

[4] Center of Excellence in Elephant and Wildlife Research, Chiang Mai University, Chiang Mai 50100, Thailand

[5] National Elephant Institute, Forest Industry Organization, Lampang 52190, Thailand

[6] Department of Companion Animals and Wildlife Clinics, Faculty of Veterinary Medicine, Chiang Mai University, Chiang Mai 50100, Thailand

¶ Contributed equally to this work

[*] Corresponding author

E-mail: kidsadagon.p@cmu.ac.th (KP)

Supplementary information

S1 Table: Blood profile of EEHV-negative elephants used as control in this study.

**EEHV negative**		**Ref. range***
No. of animals	217	
Mean age [year-old]	7.9	
Mean RBC count [x10^6 cells/µL]	3.49	2.5-5
Mean PCV [%]	34.91	30-40
Mean platelet count [x10^3 cells/µL]	447.18	200-600
Mean total serum protein [g/dL]	7.79 (168/217)	6-8
Albumin [g/dL]	3.05 (168/217)	1.5-3.5
Globulin [g/dL]	4.74 (168/217)	3.7-6.5
Mean fibrinogen [mg/dL]	390.76 (65/217)	100-400

* Mikota, S. K. (2006b). Hemolymphatic system. In: Biology, medicine, and surgery of elephants.

S2 Table: EEHVs viral loads in various organ tissues of fatal cases quantified by quantitative PCR. The EEHV genomes were normalized to total nucleic acid from the respective tissues.

Genotypes	Viral genome copies (vgc/100 ng of DNA input) in various tissues					
	Heart	Kidneys	Liver	Lungs	Spleen	Intestines
**EEHV1A**						
Pol1	$3.15x10^7$	$3.16x10^4$	$2.24x10^6$	$9.4x10^3$	$2.36x10^4$	$4.15x10^4$
**EEHV4**						
Ter3/4	$3.94x10^8$	$7.46x10^7$	$7.75x10^4$	$5.35x10^8$	$1.02x10^8$	$2.51x10^8$
**EEHV1A/4**						
Pol1	$9.69x10^2$	$5.58x10^3$	$1.08x10^4$	$2.11x10^3$	$1.86x10^4$	$3.1x10^4$
Ter3/4	$3.39x10^7$	$3.67x10^7$	$1.23x10^8$	$1.84x10^6$	$1.83x10^8$	$3.17x10^8$

S1 Figure. Representative histopathological photomicrographs of vascular lesions in EEHV-HD cases.

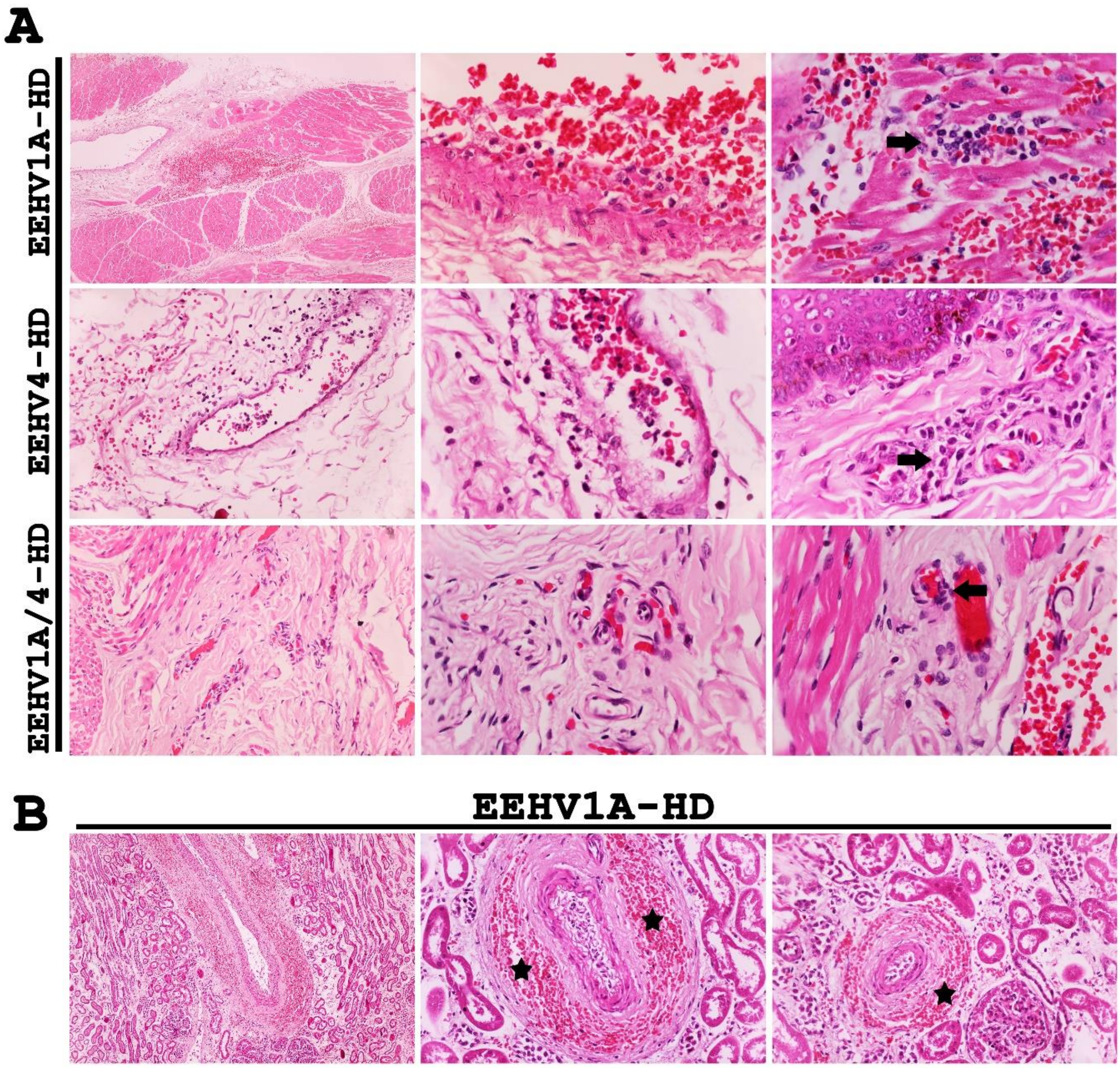

(**A**) Elephants that died due to EEHV infection showed the predominantly histopathological lesions of perivascular edema and fibrinonecrotizing or lymphohistiocytic (arrows) inflammation of the small blood vessels in the EEHV1A-HD, EEHV4-HD or EEHV1A/4-HD cases. (**B**) Medium and large blood vessels of EEHV1A-HD were observed to show a limited inflammation and hemorrhaging in the vasa vasorum of the tunica adventitia and media (stars).
